# Inhibitory Control Processes and the Strategies That Support Them during Hand and Eye Movements

**DOI:** 10.3389/fpsyg.2016.01927

**Published:** 2016-12-09

**Authors:** Lauren M. Schmitt, Lisa D. Ankeny, John A. Sweeney, Matthew W. Mosconi

**Affiliations:** ^1^Clinical Child Psychology Program, Shiefelbusch Institute for Life Span Studies, University of Kansas, LawrenceKS, USA; ^2^Department of Psychology, University of Denver, DenverCO, USA; ^3^Department of Psychiatry and Behavioral Neuroscience, University of Cincinnati, CincinnatiOH, USA

**Keywords:** Stop Signal test, inhibitory control, response inhibition

## Abstract

**Background and Aims:** Adaptive behavior depends on the ability to voluntarily suppress context-inappropriate behaviors, a process referred to as response inhibition. Stop Signal tests (SSTs) are the most frequently studied paradigm used to assess response inhibition. Previous studies of SSTs have indicated that inhibitory control behavior can be explained using a common model in which GO and STOP processes are initiated independent from one and another, and the process that is completed first determines whether the behavior is elicited (GO process) or terminated (STOP process). Consistent with this model, studies have indicated that individuals strategically delay their behaviors during SSTs in order to increase their stopping abilities. Despite being controlled by distinct neural systems, prior studies have largely documented similar inhibitory control performance across eye and hand movements. Though, no existing studies have compared the extent to which individuals strategically delay behavior across different effectors is not yet clear. Here, we compared the extent to which inhibitory control processes and the cognitive strategies that support them during oculomotor and manual motor behaviors.

**Methods:** We examined 29 healthy individuals who performed parallel oculomotor and manual motor SSTs. Participants also completed a separate block of GO trials administered prior to the Stop Signal tests to assess baseline reaction times for each effector and reaction time increases during interleaved GO trials of the SST.

**Results:** Our results showed that stopping errors increased for both effectors as the interval between GO and STOP cues was increased (i.e., stop signal delay), but performance deteriorated more rapidly for eye compared to hand movements with increases in stop signal delay. During GO trials, participants delayed the initiation of their responses for each effector, and greater slowing of reaction times on GO trials was associated with increased accuracy on STOP trials for both effectors. However, participants delayed their eye movements to a lesser degree than their hand movements, and strategic reaction time slowing was a stronger determinant of stopping accuracy for hand compared to eye movements. Overall, stopping accuracies for eye and hand movements were only modestly correlated, and the time it took individuals to cancel a response was not related for eye and hand movements.

**Discussion and Conclusion:** Our findings that GO and STOP processes are independent and that individuals strategically delay their behavioral responses to increase stopping accuracy regardless of effector indicate that inhibitory control of oculomotor and manual motor behaviors both follow common guiding principles. Yet, our findings document that eye movements are more difficult to inhibit than hand movements, and the timing, magnitude, and impact of cognitive control strategies used to support voluntary response inhibition are less robust for eye compared to hand movements. This suggests that inhibitory control systems also show unique characteristics that are behavior-dependent. This conclusion is consistent with neurophysiological evidence showing important differences in the architecture and functional properties of the neural systems involved in inhibitory control of eye and hand movements. It also suggests that characterizing inhibitory control processes in health and disease requires effector-specific analysis.

## Introduction

Voluntary inhibition of unwanted behavioral responses is a central component of executive control, and it is necessary for flexibly adapting behavior to changing environmental demands. Determining the cognitive and neural mechanisms involved in voluntary response inhibition is critical for understanding behavioral development in health as well as in psychiatric, developmental and neurological conditions associated with inhibitory control impairments (Oosterlaan et al., 1998; Aron et al., 2003; Gauggel et al., 2004; Mirabella et al., 2008, 2012; Harris et al., 2009; Mosconi et al., 2009; Lipszyc and Schachar, 2010). Studies have indicated that inhibitory control processes operate under relatively uniform guiding principles across different behaviors and effectors. For example, successful response inhibition has been modeled as both a speed accuracy trade-off (Schouten and Bekker, 1967) and a race between going and stopping processes (Logan and Cowan, 1984). Yet, the cognitive and brain processes supporting response inhibition still are not fully understood, especially with regard to the degree to which they vary across different effector systems.

Stop Signal tests (SSTs) are the most widely used laboratory measure of response inhibition (Vince, 1948; Lappin and Eriksen, 1966; Logan et al., 1984; Logan, 1994; Bissett and Logan, 2011). In this type of task, individuals must balance the opposing demands of “GO” trials in which they are prompted to generate a rapid motor response, and interspersed, less frequently presented “STOP” trials that require them to cancel their motor response. On the latter trials, a Stop Signal cue is presented at varying time intervals after the target cue, i.e., the stop signal delay (SSD). Performance on SSTs has been modeled as a race between independent stochastic GO and STOP processes (Ollman, 1973; Logan, 1981). This “independent race model” has been used to generate an estimate of the time needed to cancel a planned movement, i.e., the stop signal reaction time (SSRT; Logan and Cowan, 1984; Logan, 1994). For the STOP process to successfully cancel a planned movement, the cumulative duration of the SSD and the SSRT must be shorter than the time it takes to complete the targeted motor response elicited by the GO cue (Logan et al., 1984; Logan, 1994; Boucher et al., 2007a). Therefore, increasing the SSD decreases the likelihood that the STOP process will be completed prior to the GO process (Logan et al., 1984).

Studies of Stop Signal performance have indicated that the race model can be applied to inhibitory control processes across multiple effectors and behaviors, including reaching movements (Mirabella et al., 2012), button presses (Logan, 1994), precision gripping (De Jong et al., 1990), foot presses (De Jong et al., 1995), and saccadic eye movements (Boucher et al., 2007a). For example, studies directly comparing eye and hand movements have documented similar SSRTs and levels of stopping across effectors (Logan and Irwin, 2000; Leung and Cai, 2007; Cai et al., 2014; Gulberti et al., 2014). Based on these behavioral findings, inhibitory control processes are often assumed to fit a common model that can be generalized across different behaviors and effectors (Logan and Irwin, 2000; Boucher et al., 2007b).

In contrast to these behavioral findings, neurophysiological evidence indicates that brain networks supporting inhibitory control of oculomotor and manual motor systems show important distinctions. Specifically, inhibition of oculomotor behaviors involves interactive excitatory and inhibitory cells within frontal eye fields, dorsal caudate nuclei, and midbrain nuclei (Leung and Cai, 2007; Cai et al., 2014). In contrast, inhibition of manual behaviors involves more distributed networks of GO and STOP mechanisms in which GO processes initiated by motor cortices may be interrupted by distinct indirect and hyperdirect frontostriatal systems involving right middle frontal gyrus, subthalamic nucleus, and pre-supplementary motor cortex (Aron and Poldrack, 2006; Leung and Cai, 2007; Cai et al., 2014). Further, eye movements are more ballistic and executed more rapidly than hand movements because they are subjected to reduced inertia or torsional effects compared to skeletomotor (e.g., hand, reaching) movements (Leigh and Zee, 2006). Distinct upper and lower motor neuron pathways involved in the inhibition of eye and hand movements may contribute to inhibitory control differences between these effectors (Heimer, 2012). For example, the timing of responses to sensory input is dramatically shorter for saccadic eye movements compared to hand movements (Wijnen and Ridderinkhof, 2007). Shorter reaction times afford a smaller time window during which eye movements can be canceled, or for the STOP processes to win out over the GO process. Overall, neural systems supporting manual motor behavioral control appear to be more widely distributed and slower acting than those supporting oculomotor processes. In contrast to results from prior behavioral studies, these neurophysiological findings suggest that distinct inhibitory control processes may be involved in stopping oculomotor and manual motor behaviors.

A closer analysis of prior SST behavioral studies provides evidence that there may be important task-dependent differences in stopping mechanisms for oculomotor and manual motor behaviors. For example, individuals had reduced abilities to stop eye compared to hand movements are seen only when responses are peripherally cued (Logan and Irwin, 2000) or stop cues were presented at longer SSDs (Boucher et al., 2007b). This suggests that increasing the reflexivity of the target behavior (e.g., by inducing eye movements away from versus toward the center; Posner et al., 1980) or increasing the difficulty of stopping the target behavior (i.e., by increasing SSD) may have more deleterious effects on oculomotor compared to manual motor stopping. Thus, determining stimulus characteristics associated with behavioral response differences across effectors will be important for clarifying effector-specific inhibitory control processes.

One additional difference between oculomotor and manual motor stopping behavior may be the extent to which these distinct systems involve top-down cognitive control strategies. Prior SST studies have shown that participants strategically delay the onset of their oculomotor and manual motor responses in order to determine if a STOP cue will be presented, and that these reaction time adjustments are associated with improved stopping ability (Emeric et al., 2007; Verbruggen and Logan, 2009; Bissett and Logan, 2011). *Proactive* response delays involve slowing the initiation of a behavior based upon estimates of the probability of receiving a Stop Signal (Logan and Burkell, 1986; Boucher et al., 2007b; Mirabella et al., 2008; Verbruggen and Logan, 2009; Bissett and Logan, 2011; Federico and Mirabella, 2014). In contrast, *reactive* response delays are based upon the type and accuracy of the preceding trial (Rieger and Gauggel, 1999; Cabel et al., 2000; Kornylo et al., 2003; Mirabella et al., 2006; Emeric et al., 2007; Verbruggen and Logan, 2008, 2009). For example, participants have been shown to *proactively* delay their responses more when STOP trials are increased in frequency (Logan et al., 1984), and they *reactively* delay their responses more following STOP compared to GO trials (Rieger and Gauggel, 1999; Verbruggen and Logan, 2008, 2009). These strategic biases may be more difficult for highly reflexive behaviors such as peripherally cued eye movements, though direct comparisons of the impact of these strategies on eye and hand stopping have not been performed. Such comparisons may provide important insights into the cognitive operations that support response inhibition of different effectors, the mechanisms contributing to the development of inhibitory control in health, and the cognitive and brain processes that are impacted in different neurodevelopmental and neurological conditions associated with broader deficits of behavioral response inhibition.

In the present study, participants completed SSTs of saccadic eye movements and manual button pressing designed to be as similar as possible. Our primary aim was to characterize differences in the abilities to stop peripherally cued oculomotor and manual motor behaviors over a large range of SSDs. It was hypothesized that eye movements would be more difficult to inhibit than hand movements, particularly when the task became more difficult as SSDs were increased. We also aimed to determine the extent to which strategic reaction time adjustments were used to support oculomotor and manual motor stopping. Given the increased level of reflexivity of eye compared to hand movements, it was expected that reaction time adjustments would be smaller and would have less effect on stopping ability during the oculomotor compared to the manual motor SST. Last, we aimed to determine the degree to which oculomotor and manual motor inhibitory control were related. Based on evidence that the neural systems involved in oculomotor and manual motor stopping show distinct functional characteristics and anatomical distribution (Leung and Cai, 2007; Cai et al., 2014), we postulated that oculomotor and manual motor inhibitory control abilities would show only modest interrelationships. To determine whether inhibitory control processes were related to general cognitive abilities, we also examined the relationships between Stop Signal performance and IQ.

## Materials and Methods

### Participants

Twenty-nine healthy, right-handed individuals (15 male and 14 female) between 15–35 years of age (mean = 25; *SD* = 6) performed oculomotor and manual motor SSTs. Handedness was confirmed using the Annett Hand Preference Questionnaire (Annett, 1970). The lower age limit was determined based upon our prior work showing that inhibitory control abilities reach adult levels by age 15 years old (Luna et al., 2004). Tests including only GO trials also were administered to determine baseline reaction times for each effector for each individual. Participants were free of caffeine for 24 h prior to testing and they did not use nicotine for 1 h prior to testing. Among our participants, thirteen individuals identified as not drinking caffeine regularly, 13 as light drinkers (1–2 cups per day), and three as moderate drinkers (3–4 cups per day). With regard to daily nicotine intake, 26 participants identified as non-smokers and three as light smokers (0–10 cigarettes). Because our participants were not able consume caffeine or nicotine prior to testing, it is possible that they may have experienced acute withdrawal during the study; however, no participants reported signs of acute withdrawal during testing. All adult participants provided written consent and minors provided assent in addition to written consent from their legal guardian.

### Apparatus and Stimuli

For both oculomotor and manual motor tests, visual stimuli (i.e., white dot) subtending 0.5–1° of visual angle were presented on a black monitor in the horizontal plane at eye level. A centrally located white crosshair was presented prior to each trial. During eye movement testing, all participants sat in a darkened black room 60 cm from a 101.6 cm anti-glare LCD screen monitor with a resolution of 1920 × 1060. Participants were positioned in a chin rest and eye movements were monitored using infrared sensors that detected saccades with amplitudes ≥0.20–0.25° (Model 310, Applied Science Laboratories, Inc, Bedford, MA, USA). Fixation of static targets across the horizontal plane was used to calibrate eye movement recordings. Blinks were monitored using electrodes placed above and below the left eye linked to an AC-coupled bioamplifier. Eye movement data were digitized at 500 Hz with a 12 bit A/D converter (DI-720 from Dataq Instruments, Akron, OH, USA). Digital finite impulse response filters with non-linear transition bands were applied prior to analyses of the eye movement data. For manual motor testing, participants were seated in front of a 50.8 cm monitor with a resolution of 1680 × 1050 (Dell 1905FP). Participants used a custom-made button box that recorded finger presses through a USB port with a sampling rate of 125 Hz. Stimuli for oculomotor and manual motor testing were presented using Adobe Flash software (Flash MX Actionscript 2).

### Procedure

#### Oculomotor Stop Signal Test (Interleaved GO and STOP Trials)

**FIGURE 1:**
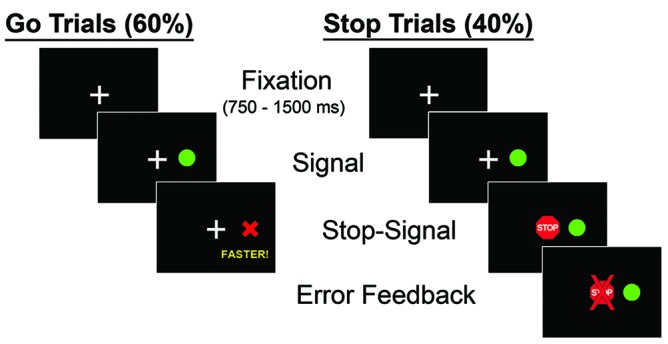
**Sequence of stimulus presentation during the oculomotor and manual motor Stop Signal tests.** Following fixation, “GO” and “STOP” trials were pseudorandomly presented. During GO trials, subjects responded to a peripheral target appearing in either the right or left hemifield by looking toward the target (oculomotor) or pressing the corresponding button (manual motor). During STOP trials, a central STOP cue was presented at a variable delay following the onset of the GO cue. Participants were instructed to inhibit their response when the STOP cue appeared. GO trials constituted approximately 60% of task trials, but every third incorrect GO trial was repeated in order to encourage participants to respond throughout the task.

Trials requiring saccadic eye movements began while participants fixated their gaze on a central crosshair. Participants had to maintain fixation within +3° from center for a random duration of 750–1500 ms before the peripheral stimulus appeared, marking the onset of each trial (**Figure 1**). During GO trials of the SST, a green circular target appeared 12° to the right or left of center, and participants were instructed to shift their gaze to the target as quickly as possible. Saccade onset was defined at the time-point at which eye velocity exceeded 30°/s. During oculomotor testing, participants were instructed to keep their hands resting comfortably on the table in front of them.

In order to ensure that participants responded to GO trials without waiting indefinitely to determine if a STOP cue would be presented, two steps were taken. First, if a response to GO trials did not occur within 650 ms, a red “X” immediately appeared in place of the green target, along with the word “faster” below the “X” for 2500 ms in order to ensure that participants processed feedback. Second, every third GO trial in which subjects did not respond within 650 ms was repeated at a later random trial during the task.

During ‘STOP’ trials, a red STOP cue replaced the central fixation cue at varying SSDs after the GO cue was presented. Participants were instructed to avoid shifting their eye gaze when the STOP cue appeared. SSDs were sampled continuously in 13.33 ms intervals (matching the monitor refresh rate of 75 Hz) between 50–200 ms. For each of the 11 SSDs, 4–5 trials were included. Eight participants were tested on a separate monitor with a different refresh rate (120 Hz) and therefore completed trials with SSDs sampled continuously in 8.33 ms intervals. For each of these 18 SSDs, three to four trials were included. The monitor used for testing was switched for the final eight participants due to technical issues; however, analyses performed using monitor as a covariate did not alter the results, so testing data from both monitors were combined.

The order of SSDs was randomized, and different trial types were presented in a pseudorandomized order; no more than three consecutive trials of the same type (GO or STOP) were administered. If a participant responded on a STOP trial, a red ‘X’ was displayed centrally for 1000 ms immediately following their error. Incorrect STOP trials were not repeated. Four blocks of 63 trials [38 GO (60%) and 25 STOP (40%)] were administered consistent with GO:STOP trial ratios used in prior studies (Ethridge et al., 2014). Each block of trials was followed by 10 s of rest. Reaction times for correct GO trials and the proportion of STOP trials in which subjects correctly inhibited their response were examined. SSRTs also were examined by modeling the SSD at which stopping accuracy equaled 50% (p50) for each participant, and then subtracting this value from each individual’s mean reaction time for GO trials (i.e., SSRT = mean reaction time – p50; Logan and Cowan, 1984; Logan, 1994; Logan et al., 1997; Aron and Poldrack, 2006; Boucher et al., 2007a).

#### Manual Motor Stop Signal Test (GO and STOP Trials)

The manual motor SST was designed to parallel the oculomotor SST as closely as possible. Task stimuli and timing were similar, and we chose to study a manual behavior (i.e., button pressing) that was as rapid and simple as possible to closely match the reflexive nature of saccadic eye movements. During this test, participants rested their thumbs on left and right buttons corresponding to the locations of the peripheral targets. They were instructed to press the correct button as quickly as possible on all GO trials while maintaining their fixation on the central crosshair. STOP trial SSDs were sampled between 50–283 ms to match the refresh rate of the monitor specifically used for the manual motor version of this task (60 Hz, or every 16.67 ms). Seven to eight trials were included for each of the 14 SSDs. The maximum SSD for the manual motor SST was higher (283 ms) than that for the oculomotor test (200 ms) based on prior studies showing continued ability to stop manual responses at these longer SSD intervals (Logan and Irwin, 2000). Participants were randomly assigned to receive either eye movement or manual motor testing first.

#### Baseline Reaction Time Tests (Blocked GO Trials)

In order to assess participants’ reaction times during a condition in which they would have no bias to strategically delay their responses, oculomotor and manual motor baseline reaction time tasks including only GO trials were administered. Baseline reaction time tests included 30 GO trials (15 rightward, 15 leftward) presented in the same format as GO trials in the SSTs. A small number of baseline trials were used due to minimal variability in reaction times of basic saccadic movements and manual button presses. These baseline reaction time tasks always preceded the SST of the same effector.

#### Cognitive Assessment

The Wechsler Abbreviated Scales of Intelligence (WASI; Wechsler, 1999) were administered so that the relationship between general intellectual functioning and Stop Signal performance (e.g., % correct STOP trials, SSRT, p50, reaction time delay) could be determined for each effector.

### Statistical Analyses

#### Aim 1

We conducted *t*-tests to compare eye and hand performance on primary outcome variables including the percentage of accurate STOP trials, p50, and SSRT. Shapiro Wilk’s *W* Tests were used to assess for normality of the distributions of all outcome variables. Mixed-effects logistic regression was used to examine how stopping accuracy (% correct cancelations on STOP trials) varied as a function of SSD as done in prior studies (Ethridge et al., 2014).

#### Aim 2

In order to compare strategic reaction time slowing in eyes and hands, a 2 (effector: eye vs. hand) × 2 (task: baseline vs. Stop Signal task) ANOVA was conducted to predict reaction time. Pearson correlations were performed to determine relationships between reaction time slowing and percentage of accurate STOP trials, p50, and SSRT within each effector. Fisher *Z* transformations were used to compare the strength of these relationships for eye and hand movements.

#### Aim 3

To determine the extent to which oculomotor and manual motor processes were related to each other, Pearson correlations were used to assess associations between the percentage of correct STOP trials, p50, SSRT and reaction times across oculomotor and manual motor tests. Also, in order to clarify the relationship between Stop Signal performance and general cognitive ability, we calculated Pearson correlations between Stop Signal performance and Full Scale IQ estimates from the WASI.

## Results

### Oculomotor and Manual Motor Stopping Abilities

**FIGURE 2:**
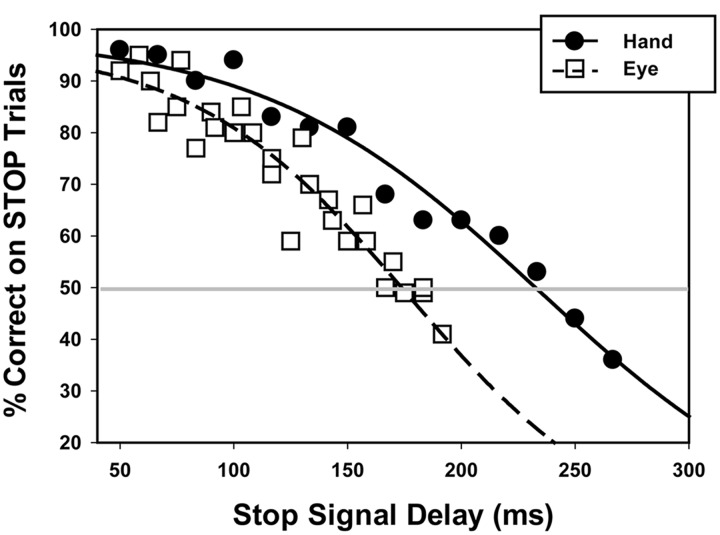
**STOP trial accuracy across various Stop Signal Delays during the oculomotor (dashed line) and manual motor (solid line) tasks.** Data points represent mean performance across participants at each Stop Signal Delay for the eye (open square) and hand (closed circle). The gray horizontal line is shown to indicate the Stop Signal Delay at which individuals had a 50% probability of accurately inhibiting oculomotor (178 ms) and manual motor responses (227 ms). *p*50 values shown here are the means across subjects for each effector.

**Table 1** shows stopping accuracy for the SSTs and reaction times on GO trials for Stop Signal and baseline reaction time tests. All variables were normally distributed (ω^2^’s > 0.96, *p*’s > 0.24). As predicted, our mixed-effects logistic regression revealed that stopping accuracy decreased with increasing SSDs for both oculomotor (*Z* = -7.55, *p* < 0.001) and manual motor testing (*Z* = -10.79, *p* < 0.001; **Figure 2**). The SSD at which each subject’s accuracy on STOP trials equaled 50% was greater for manual than oculomotor responses [hand = 227 ms (*SD* = 56); eye = 178 ms (*SD* = 71); *t*(24) = 3.09; *p* = 0.005], consistent with the hypothesis that participants show a faster decline in performance with increasing SSD for eye compared to hand movements. Overall, participants were more successful inhibiting manual responses than oculomotor responses when analyses were restricted to SSDs used in both hand and eye movement tasks (*Z* = 2.63, *p* = 0.009). SSRT was longer for manual compared to oculomotor responses [hand = 221(*SD* = 43); eye = 146(*SD* = 75); *t*(24) = 4.49, *p* < 0.001]. The percentage of correctly inhibited STOP trials was not related to SSRT for either oculomotor or manual motor performance [eye *r*(24) = 0.11, *p* = 0.30, hand *r*(29) = -0.25, *p* = 0.20).

**Table 1:** Oculomotor and manual motor stopping performance and GO reaction times during baseline and Stop Signal tests.

	Oculomotor	Manual Motor	*p-value*
**Stopping performance**	
Accuracy (%correct)^1^	62 (14)	84 (11)	<0.001
SSRT (ms)	146 (75)	221 (43)	<0.001
p50	178 (71)	227 (56)	0.005
**Reaction Time in ms**	
GO baseline trials	208 (34)	303 (32)	<0.001
GO SST trials	327 (42)	448 (31)	<0.001


*mean (SD)*

*^1^only trials with SSD <200 ms were included so that oculomotor and manual motor performance could be directly compared*

*ms, milliseconds; SSRT, stop signal reaction time.*

### Manual Motor and Oculomotor Reaction Time

**FIGURE 3:**
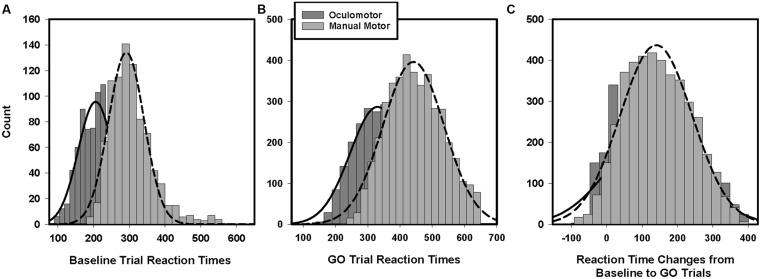
**Histograms of baseline and GO trial reaction times.** Manual motor reactions times during the baseline trials **(A)** and GO trials **(B)** are longer than oculomotor reaction times. Manual motor reaction times also were slowed to a greater degree than oculomotor reaction times from baseline to GO trials **(C)**.

Manual reaction times were longer than saccade reaction times during baseline GO trials [**Figure 3A**; hand = 303 ms (*SD* = 32); eye = 208 ms (*SD* = 34); *t*(27) = 10.43, *p* < 0.001]. Manual reaction times also were longer than saccade reaction times on Stop Signal task GO trials [**Figure 3B**; hand = 448 ms (*SD* = 31); eye = 327 ms (*SD* = 42); *t*(27) = 18.85, *p* < 0.001]. The degree to which manual reaction times were slower than oculomotor reaction times was greater for Stop Signal GO trials compared to baseline trials [Task × Effector interaction: *F*(1,25) = 7.81; *p* = 0.01].

During the SSTs, participants slowed both their manual and oculomotor reaction times relative to their baseline reaction times [hand: *F*(1,28) = 247.78; *p* < 0.001; eye: *F*(1,28) = 338.40; *p* < 0.001]. They slowed their manual reaction times more than their eye movement reaction times [**Figure 3C**; hand = +145 ms (*SD* = 45); eye = +119 ms (*SD* = 33); *F*(1,28) = 9.71; *p* < 0.01]. The amounts that individuals strategically slowed their reaction times during manual motor and oculomotor SST GO trials were correlated [**Table 2**; *r*(27) = 0.44, *p* = 0.02]. Participants’ reaction times during manual GO trials were not related to their baseline reaction times [*r*(30) = -0.03, *p* = 0.89]. In contrast, Stop Signal oculomotor reaction times were related to baseline reaction times [*r*(27) = 0.65, *p* < 0.01]. Manual motor and oculomotor reaction times during baseline GO trials were not related to each other [*r*(30) = -0.14, *p* = 0.45], but reaction times for manual and oculomotor responses during GO trials were related [*r*(27) = 0.57, *p* = 0.002].

**Table 2:** Relationships between oculomotor and manual motor reaction times and stopping accuracy.

		Oculomotor		Manual motor		
			
		GO RT	STOP trial accuracy	Baseline RT	GO RT	STOP trial accuracy
	Baseline GO RT	0.65^∗∗∗^	-0.10	-0.14	0.11	0.18
**Oculomotor**	GO RT	–	0.39^∗^	-0.21	0.39^∗^	0.44**
	STOP trial accuracy		–	-0.25	0.20	0.41*
**Manual motor**	Baseline GO RT			–	-0.03	-0.07
	GO RT				–	0.87***


*RT, reaction time.*

*^∗^*p* < 0.05; ^∗∗^*p* < 0.01; ^∗∗∗^*p* < 0.001*

**FIGURE 4:**
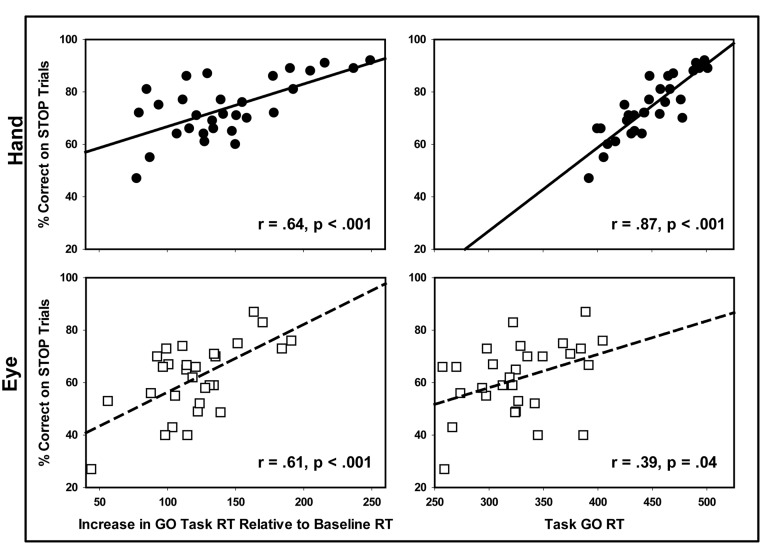
**Relationships between reaction times for Stop Signal test GO trials and STOP trial accuracy for the eye and hand.** Longer GO trial reaction times during the Stop Signal test were associated with increased rates of stopping accuracy for both eye and hand movements. The strength of this relationship was greater for hand than eye movements.

As seen in **Figure 4**, greater reaction time slowing from baseline to GO trials was associated with an increased percentage of successful STOP trials for both the manual [*r*(27) = 0.64, *p* < 0.001] and oculomotor tests [*r*(27) = 0.61, *p* < 0.001]. GO trial reaction times also were associated with an increased percentage of successful STOP trials on both manual [*r*(27) = 0.87, *p* < 0.001] and oculomotor tasks [*r*(27) = 0.39, *p* = 0.04]; however, the relationship was stronger for manual compared to eye movement responses (*Z* = 3.32, *p* < 0.01). Thus, slower reaction time during the SST was a greater predictor of stopping success for manual compared to ocular motor responses. Baseline reaction times were not predictive of either oculomotor or manual stopping accuracy [hand *r*(29) = -0.16, *p* = 0.40; eye *r*(28) = -0.27, *p* = 0.28]. We found no relationship GO trial reaction time slowing and SSRTs for either the eye or hand, consistent with the hypothesis that GO and STOP processes are independent [eye *r*(24) = 28,*p* = 0.19; hand *r*(29) = -0.12, *p* = 0.53].

**FIGURE 5:**
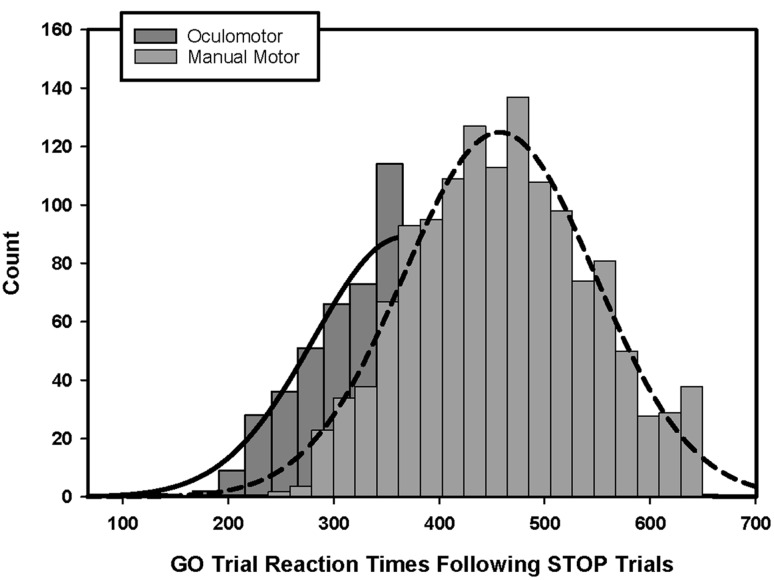
**Histogram of reaction time slowing on GO trials following STOP trials.** Manual motor reaction times were slowed to a greater degree compared to oculomotor reaction times on GO trials when preceded by STOP trials.

For both the manual and oculomotor Stop Signal tasks, reaction time slowing during GO trials varied according to the type of the preceding trial. Participants slowed their reaction times following STOP trials more than following GO trials [**Table 3**; hand: *F*(1,28) = 5.63, *p* = 0.03; eye: *F*(1,28) = 79.49, *p* < 0.001], and this difference was greater for oculomotor compared to manual responses [**Figure 5**; Effector × Previous Trial Type interaction: *F*(1,28) = 5.28, *p* = 0.03]. During the manual task, reaction times following STOP [*r*(27) = 0.81, *p* < 0.001] and GO trials [*r*(27) = 0.56, *p* < 0.001] were both associated with increased accuracy on STOP trials. However, for eye movements, increased stopping accuracy was only related to increases in reaction times following GO trials [*r*(27) = 0.46, *p* = 0.01], and not following STOP trials [*r*(28) = 0.27, *p* = 0.15].

**Table 3:** GO trial reaction times during the Stop Signal test for eye and hand movements separated by whether trials followed a GO trial or a STOP trial and by whether trials followed accurate or inaccurate trials on both GO and STOP trials.

	Previous trial type		
		
	GO trial	STOP trial	STOP-GO Difference
Oculomotor RT	299 (45)	353 (45)	+54 ms
Manual motor RT	429 (49)	450 (39)	+21 ms

	**Outcome of previous trial**		
		
	**Accurate**	**Inaccurate**	**Inaccurate-Accurate Difference**

**Post-GO trials**			
Oculomotor RT	317 (43)	312 (80)	-5 ms
Manual motor RT	441 (47)	438 (63)	-3 ms
**Post-STOP trials**			
Oculomotor RT	366 (41)	374 (81)	+8 ms
Manual motor RT	452 (42)	486 (42)	+34 ms


*mean (SD)*

The extent to which reaction times were adjusted during GO trials also depended on the accuracy of the previous response. Participants slowed their responses to a greater degree following inaccurate STOP trials compared to accurate STOP trials [*F*(1,25) = 7.83, *p* = 0.01]. When comparing oculomotor and manual motor responses, there was no difference in the degree to which participants preferentially slowed their responses for inaccurate versus accurate STOP trials (*p* = 0.11). Increases in reaction time following STOP trial failures were associated with increases in manual stopping accuracy [*r*(27) = 0.50, *p* = 0.01] but not oculomotor stopping accuracy [*r*(26) = 0.17, *p* = 0.41]. Participants did not alter their response times according to the accuracy of previous GO trials (*p*’s > 0.70).

### Inter-relationship between Oculomotor and Manual Stop Signal Test Performance

Accuracy for manual motor and oculomotor STOP trials was modestly correlated [**Table 2**; *r*(27) = 0.41, *p* = 0.03]. Manual motor and oculomotor SSRTs were not significantly correlated with each other [*r*(24) = 0.11, *p* = 0.66]. The SSDs at which participants’ eye and hand stopping accuracy equaled 50% also were not related [*r*(25) = 0.17, *p* = 0.41].

### Relationship between Stop Signal Test Performance and General Intellectual Ability

**FIGURE 6:**
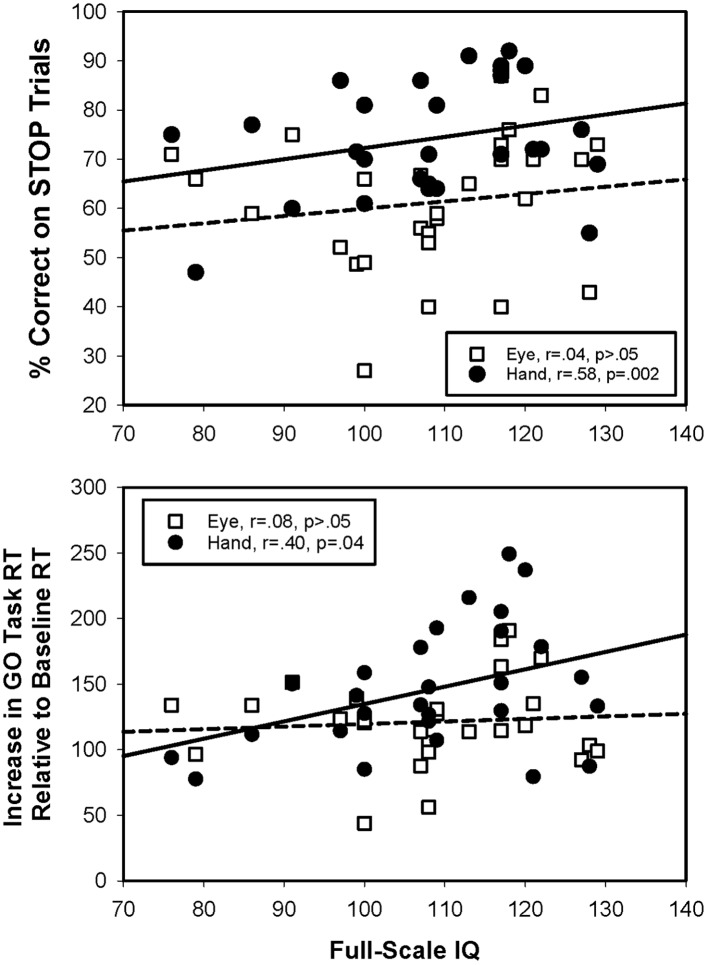
**Relationships between Stop Signal test performance and general intellectual ability.** Greater general intellectual ability was associated with greater stopping accuracy for hand, but not for eye movements. Also, greater general intellectual ability was associated with greater slowing of reaction times from baseline GO trials to Stop Signal GO trials for the hand, but not the eye.

Greater stopping accuracy [*r*(28) = 0.58, *p* < 0.01] and greater delays [*r*(28) = 0.40, *p* = 0.04] in GO trial responses during the manual motor SST were associated with higher Full Scale IQ (**Figure 6**). No significant relationships between IQ and oculomotor stopping accuracy [*r*(26) = 0.04, *p* = 0.86] or slowing were seen [*r*(27) = 0.08, *p* = 0.69]. SSRTs were not related to IQ for either effector [eye: *r*(22) = 0.26, *p* = 0.27; hand: *r*(27) = 0.34, *p* = 0.08].

## Discussion

The present study documents three key differences between inhibitory control processes involved in stopping oculomotor and manual motor behaviors. First, eye movements were more difficult to inhibit than manual responses, and oculomotor stopping ability deteriorated more rapidly than manual motor stopping ability as SSDs increased. Second, the extent to which participants delayed their reaction times during GO trials relative to baseline trials was greater for hand than eye movements, and it was more strongly associated with stopping ability for hand compared to eye movements. Third, stopping abilities for eye and hand movements were only modestly correlated, and oculomotor and manual motor SSRTs were not related. Overall, these findings indicate that oculomotor responses are under less volitional control and are less amenable to strategic adjustments of reaction timing than manual motor behaviors. Our finding that manual motor but not oculomotor stopping ability is related to general cognitive ability provides further evidence that response inhibition of these two effector systems involves different underlying cognitive processes.

### Oculomotor and Manual Motor Stopping Abilities

Our finding that individuals were better able to inhibit manual compared to oculomotor responses likely reflects the more reflexive nature and reduced inertia of eye movements relative to limb movements (Leigh and Zee, 2006; McDowell et al., 2008). Reflexive movements are made in direct response to novel external stimuli and primarily involve sensorimotor processes, whereas volitional movements involve both sensorimotor and higher-order cognitive processes. Our results are consistent with several previous reports showing higher rates of successful manual motor compared to oculomotor stopping during SSTs (Logan and Irwin, 2000; Boucher et al., 2007b) as well as alternative inhibitory control paradigms (Bekkering et al., 1994). Yet, the majority of previous studies suggest that oculomotor and manual motor stopping abilities are similar during SSTs (Logan and Irwin, 2000; Boucher et al., 2007b; Leung and Cai, 2007; Cai et al., 2014; Gulberti et al., 2014). Differences between these latter studies and ours highlight the strong influence of task-related factors that show greater impact on stopping ability of eye compared to hand movements. For example, Boucher et al. (2007b) documented that oculomotor and manual motor stopping are comparable when target behaviors are elicited by visually presented peripheral cues at shorter SSDs. Yet, they also found that oculomotor stopping is more difficult than manual motor stopping when target behaviors are elicited by peripheral cues at *longer* SSDs, consistent with our results. Similarly, Logan and Irwin (2000) documented comparable levels of oculomotor and manual stopping ability when central GO and STOP cues were used, but worse oculomotor compared to manual motor performance when peripheral GO and STOP cues were used. Peripheral visual cues evoke more reflexive responses than central visual cues, and reaction times for eyes are faster than for hands (Posner et al., 1980; Wijnen and Ridderinkhof, 2007). Thus, eye movements are more difficult to inhibit because faster oculomotor reaction times would reduce the probability of oculomotor STOP processes finishing prior to the completion of the GO process. This is be particularly true at longer SSDs, when the STOP process begins later.

### Reaction Time Adjustments during Manual Motor and Oculomotor Inhibitory Control

Consistent with previous studies, we found that GO trial reaction times increased during the SSTs compared to baseline suggesting that individuals delay the start of the GO processes when task demands are uncertain (Logan and Burkell, 1986; Boucher et al., 2007a; Mirabella et al., 2008; Verbruggen and Logan, 2009; Bissett and Logan, 2011; Federico and Mirabella, 2014). Strategic delays in reaction timing also were associatd with improved stopping ability as has been documented previously (Emeric et al., 2007; Verbruggen and Logan, 2009; Bissett and Logan, 2011). We document the novel finding that strategic adjustments to reactions times are greater for hand compared to eye movements and more effective in improving stopping ability for hand compared to eye movements (predicting 64 vs. 16% of variance in STOP trial accuracy). These findings suggest that the increased capacity to stop hand compared to eye movements may reflect differences in the abilities to strategically delay hand and eye movements in uncertain conditions. Increases in reaction times prolong the finishing time of the GO process, thus providing increased opportunity for STOP processes to interrupt manual compared to oculomotor responses. Therefore, we propose that differences in the abilities to stop unwanted oculomotor and manual motor behaviors may reflect underlying differences in the degree to which reaction time adjustments can be made for different effectors.

Consistent with this idea, it has been hypothesized that reflexive movements driven by external sensory stimuli and occurring on a more rapid time scale show less amenability to top-down control processes (Leigh and Zee, 2006; Wijnen and Ridderinkhof, 2007; McDowell et al., 2008). Saccades are faster to initiate than limb movements due to their reduced inertia and torsional effects, and manual movements involve greater mechanical loads and a higher degree of inertia (Leigh and Zee, 2006). Further, eye movements cannot be interrupted once they have been initiated, whereas hand movements may be interrupted at multiple stages, including both prior to and following the initiation of action potential firing in the activated muscle (De Jong et al., 1990; Scangos and Stuphorn, 2010). Once an oculomotor response has been initiated, there is a so-called “point of no return” in which responses will be executed regardless of top-down processes attempting to inhibit that motor response (Hanes and Schall, 1996; Hanes et al., 1998). In fact, during successfully inhibited eye movements, extraocular muscles do not show any increase in firing rates or amplitude (Godlove et al., 2011). These physiological findings are consistent with our behavioral data showing that oculomotor response timing is more difficult to delay than manual motor response timing, and that delaying response times has less of an impact on overall stopping ability for eye compared to manual behaviors. Further, we found that oculomotor, but not manual motor, reaction times during GO and baseline trials were associated with each other, suggesting that the underlying processes involved in strategically delaying manual reaction times are relatively independent of those processes associated with basic motor speed.

Our findings are consistent with the majority of prior reports that also have found that response timing adjustments vary according to the type and the accuracy of preceding trials (Rieger and Gauggel, 1999; Cabel et al., 2000; Kornylo et al., 2003; Mirabella et al., 2006; Emeric et al., 2007; Verbruggen and Logan, 2008, 2009). We provide novel evidence that greater response delays following inaccurate STOP trials are related to increased stopping ability for hand movements but not eye movements. These findings suggest that while subjects may delay the onset of their behavioral responses following negative feedback across motor systems, these adjustments are not sufficient to improve stopping ability for eye movements. The lack of effectiveness in oculomotor reaction time slowing following inaccurate STOP trials may reflect a lack of consistency in reaction time adjustments across trials, or the inadequacy of smaller adjustments for allowing STOP processes to interrupt rapid eye movements. As we did not see an increase in the variability of oculomotor reaction time slowing relative manual motor reaction time slowing (*p* > 0.05), it appears more likely that the magnitude of eye movement timing adjustments made following inaccurate STOP trials was too small to significantly impact stopping ability.

Consistent with previous studies, we also found that SSRTs were shorter for eye compared to hand movements (Logan and Irwin, 2000; Boucher et al., 2007b; Leung and Cai, 2007; Cai et al., 2014; Gulberti et al., 2014). Although shorter SSRTs often are interpreted as an indication of superior stopping ability, shorter SSRTs do not appear to be sufficient for canceling eye movements at a rate similar to manual movements, and they are not associated with better stopping ability. Instead, the more reflexive nature of rapid eye movements and reduced capacity for strategically delaying these movements override the ability of individuals to stop them more rapidly than hand movements once they are cued. Results indicating that eye movements show shorter SSRTs than hand movements but still are more difficult to inhibit also suggests that the use of SSRTs to quantify inhibitory control ability may not capture the multiple distinct motor and cognitive processes involved in successfully inhibiting unwanted responses.

### Brain Mechanisms Supporting Manual Motor and Oculomotor Inhibitory Control

Differences in oculomotor and manual motor Stop Signal performance likely reflect separations at both peripheral and central levels (Aron and Poldrack, 2006; Boucher et al., 2007a). Inhibition of oculomotor behaviors involves interactive excitatory and inhibitory cells within frontal eye fields, dorsal caudate nuclei, and midbrain nuclei (Hikosaka et al., 1989; Pare and Hanes, 2003; Pouget et al., 2011). More specifically, GO commands impact a pre-existing balance of interacting and reciprocally inhibitory gaze-shifting (movement) and gaze-holding (fixation) neural units at cortical and subcortical levels. Single-cell recordings show increased firing rates within movement neurons in the frontal eye fields, caudate nucleus, superior colliculus, and brainstem following a GO cue; however, after the appearance of a STOP cue, the firing rates of these cells rapidly decreases and fixation neurons within the frontal eye fields and superior colliculus increase their firing rate as part of a process that is believed to cancel the movement (Hanes et al., 1998; Schall et al., 2000; Schall, 2002). Thus, central processes controlling eye movements cannot be interrupted after movement cells have reached a firing rate above a certain threshold (Lisberger and Fuchs, 1978; Fuchs et al., 1985; Hanes et al., 1995; Lauwereyns et al., 2002; Kaneko, 2006; Jantz et al., 2013).

For hand movements, a “stopping” circuit that is distinct from the GO circuit has been proposed involving right inferior frontal cortex (IFC), the internal segment of the globus pallidus (GPi) and subthalamic nuclei (STN; Aron and Poldrack, 2006; Chambers et al., 2006; Coxon et al., 2006; Mirabella et al., 2011; Mattia et al., 2012). A ‘hyperdirect’ IFC-STN pathway also allows more rapid interruption of movement generating signals and appears to enhance action of the stopping pathway (Aron et al., 2003; Aron and Poldrack, 2006; Jahfari et al., 2011). Thus, hand movements may be canceled before the movement is fully executed via indirect processes involving pre-supplementary motor cortex and inferior frontral gyrus, or hyperdirect processes involving STN activation (Mink, 1996; Nambu et al., 2002; Aron and Poldrack, 2006), allowing for a greater time window for successful manual motor stopping compared to oculomotor stopping.

Direct comparisons of oculomotor and manual motor inhibitory control neurophysiological processes further suggest that these brain systems are spatially and mechanistically different. ERP data has suggested that manual motor inhibitory control systems show a more posterior distribution compared to oculomotor inhibitory control systems, which show a more frontomedial voltage distribution (Reinhart et al., 2012). Consistent with these findings, fMRI studies show more posterior activation of pre-supplementary and supplementary motor areas during manual motor compared to oculomotor inhibitory control tasks (Aron and Poldrack, 2006), as well as more ventral inferior frontal gyrus activation during manual compared to oculomotor stopping (Leung and Cai, 2007).

The central mechanisms involved in strategically delaying motor responses also are different for the eye and hand. Pre-supplementary motor, supplementary motor, and anterior cingulate cortices play an important role in supporting strategic timing adjustments and actively suppressing the inferior frontal gyrus-striatal pathway that controls the timing of movement initiation (Aron and Poldrack, 2006). However, cells of the inferior frontal gyrus involved in stopping manual behaviors appear to be located more ventral than those involved in stopping eye movements (Leung and Cai, 2007). Also, medial prefrontal cortices including the supplementary eye fields and dorsal portions of caudal cingulate cortex appear to be selectively involved in error monitoring during eye movement tasks in a manner parallel to medial prefrontal cortical circuits that are involved in strategic timing adjustments of hand movements (Stuphorn et al., 2010; Reinhart et al., 2012). Although our current study cannot confirm the contribution of differences at the cellular and circuitry level on our observed behavioral findings, these results suggest that cognitive processes involved in slowing responses of eye and hand movements are distinct and thus may contribute to our behavioral differences during test of eye and hand movement inhibitory control.

Although our results indicate relatively distinct inhibitory processes for the eye and hand, it is unlikely that these processes are completely separate. For example, both manual motor and oculomotor behaviors become more difficult to inhibit as SSD increases consistent with the independent race model (Logan, 1994; Boucher et al., 2007a). We also found that strategic slowing of reaction times contributed to greater stopping abilities for both hand and eye movements. Furthermore, central mechanisms responsible for inhibitory control of the eyes and hands also share common structures (Verfaellie and Heilman, 1987; Curtis et al., 2005; Aron and Poldrack, 2006; Li et al., 2006; Leung and Cai, 2007). Thus, as previously suggested by Logan and Irwin (2000), inhibition of oculomotor and manual motor responses likely follow similar guiding principles (e.g., independent race model), but are controlled by distinct neurocognitive and neurophysiological mechanisms.

While our study provides novel evidence that control processes involved in inhibiting oculomotor and manual motor behaviors are different, several limitations of this study should be noted. First, it is possible that our findings showing important differences between oculomotor and manual motor stopping systems may not generalize to all types of eye or hand movements. By testing inhibition across a wide range of SSDs, we were able to examine the maximum delays at which inhibitory control processes could effectively stop reflexive oculomotor and manual motor behaviors. It is possible that inhibitory control systems used to stop slower or more voluntary motor responses, such as self-initiated or central saccades and manual movements, may be more similar in terms of their effectiveness or timing across effectors. Further, we chose to directly compare stopping of the two movement types across the same SSDs results may have differed if SSDs were chosen relative to reaction times for eyes compared to hands, or if comparisons of reaction time delays were made relative to individual baseline reaction times. Also, because we sampled SSDs more frequently across a broader range, estimates of the probability of stopping behaviors at each SSD were based on a relatively small number of trials (3–8). This approach allowed us to sample more SSDs but also may have reduced the stability of our estimates of probability functions and SSRTs. The use of different monitors across the hand and eye tasks also may have affected the inhibition functions. For example, because the refresh rate of the eye movement monitor was greater than the hand movement monitor, SSDs were presented over smaller intervals and repeated for fewer trials. Based on the large difference in the slopes of the probability of stopping functions for the hand and eye, it appears unlikely that the difference in the monitor refresh rates could account for differences in stopping rates between hands and eyes. Also, future studies should monitor fixation during manual motor testing to insure that differences in stopping accuracy across effectors are not due to differences in attention to stimuli. Additionally, we chose to use a reduced ratio of GO:STOP trials compared to several prior studies (Logan and Irwin, 2000; Boucher et al., 2007b; Leung and Cai, 2007; Cai et al., 2014; Gulberti et al., 2014) in order to more densely sample stopping abilities and reaction time slowing after STOP trials. It remains unclear whether differences in oculomotor and manual motor stopping and slowing vary in magnitude across different GO:STOP trial ratios. Last, we chose to analyze raw delays in reaction time as opposed to delays calculated as a percentage of baseline reaction times because the two tasks utilized identical maximum reaction times and thus the same amount of slowing was allowed for both movement types. We believe this approach was more informative for direct comparisons with prior studies and in order to first determine whether eyes and hands are controlled differently under similar conditions.

## Conclusion

In summary, we document greater top-down inhibitory control over manual motor compared to oculomotor responses suggesting that the level of influence of peripheral and central stop commands on effector systems is unique to the type of movement that they control. We demonstrated that healthy individuals are more likely to inhibit an unwanted behavioral response if they strategically delay its onset. However, our findings that strategic biases in response timing are greater overall and have a greater impact on STOP trial performance for hand than eye movements suggest that top-down mechanisms controlling eye movements are less susceptible to cognitive biases used to improve performance, perhaps due to the more reflexive nature of peripheral eye movements. Analyses of the relationship between cognitive control strategies and response inhibition success, and how these relationships differ across behaviors and effectors will be important for understanding the cognitive and neural mechanisms that support behavioral response inhibition in both health and disease, and determining optimal teaching and intervention strategies for improving inhibitory control in children and patient populations.

## Ethics Statement

This study was approved by the Institutional Review Board of University of Illinois at Chicago and its procedures conformed to the Declaration of Helsinki. University of Illinois at Chicago Institutional Review Board Prior to testing, each adult participant signed an informed consent form and minors provided oral assent and their parents provided written consent. Minors were involved in this study. Minors provided oral assent and their parents provided written consent.

## Author Contributions

LS was involved in analyzing and interpreting data as well as drafting the manuscript. JS was involved in the conception and design of the experiment, providing consultation for data interpretation and critically revising the manuscript. LA was involved in collecting and analyzing the data as well as design of the experiment and initial manuscript drafts. MM provided consultation for data interpretation and was critically involved in revising the manuscript.

## Conflict of Interest Statement

JS has consulted to Takeda, Lilly and Roche and has received grant support from Janssen. All the other authors declare that the research was conducted in the absence of any commercial or financial relationships that could be construed as a potential conflict of interest.

The reviewer LC and the handling Editor declared their shared affiliation, and the handling Editor states that the process nevertheless met the standards of a fair and objective review.

## References

[B1] Annett M. (1970). A classification of hand preference by association analysis. *Br. J. Psychol.* 61 303–321. 10.1111/j.2044-8295.1970.tb01248.x 5457503

[B2] Aron A. R. Fletcher P. C. Bullmore E. T. Sahakian B. J. Robbins T. W. (2003). Stop-signal inhibition disrupted by damage to right inferior frontal gyrus in humans. *Nat. Neurosci.* 6 115–116. 10.1038/nn1003 12536210

[B3] Aron A. R. Poldrack R. A. (2006). Cortical and subcortical contributions to stop signal response inhibition: role of the subthalamic nucleus. *J. Neurosci.* 26 2424–2433. 10.1523/JNEUROSCI.4682-05.2006 16510720 PMC6793670

[B4] Bekkering H. Adam J. J. Kingma H. Huson A. Whiting H. T. (1994). Reaction time latencies of eye and hand movements in single- and dual-task conditions. *Exp. Brain Res.* 97 471–476. 10.1007/BF00241541 8187858

[B5] Bissett P. G. Logan G. D. (2011). Balancing cognitive demands: control adjustments in the stop-signal paradigm. *J. Exp. Psychol. Learn. Mem. Cogn.* 37 392–404. 10.1037/a0021800 21171806 PMC3064521

[B6] Boucher L. Palmeri T. J. Logan G. D. Schall J. D. (2007a). Inhibitory control in mind and brain: an interactive race model of countermanding saccades. *Psychol. Rev.* 114 376–397. 10.1037/0033-295X.114.2.376 17500631

[B7] Boucher L. Stuphorn V. Logan G. D. Schall J. D. Palmeri T. J. (2007b). Stopping eye and hand movements: are the processes independent? *Percept. Psychophys.* 69 785–801. 10.3758/BF03193779 17929700

[B8] Cabel D. W. Armstrong I. T. Reingold E. Munoz D. P. (2000). Control of saccade initiation in a countermanding task using visual and auditory stop signals. *Exp. Brain Res.* 133 431–441. 10.1007/s002210000440 10985678

[B9] Cai W. Cannistraci C. J. Gore J. C. Leung H. C. (2014). Sensorimotor-independent prefrontal activity during response inhibition. *Hum. Brain Mapp.* 35 2119–2136. 10.1002/hbm.22315 23798325 PMC4888978

[B10] Chambers C. D. Bellgrove M. A. Stokes M. G. Henderson T. R. Garavan H. Robertson I. H. (2006). Executive “brake failure” following deactivation of human frontal lobe. *J. Cogn. Neurosci.* 18 444–455. 10.1162/jocn.2006.18.3.444 16513008

[B11] Coxon J. P. Stinear C. M. Byblow W. D. (2006). Intracortical inhibition during volitional inhibition of prepared action. *J. Neurophysiol.* 95 3371–3383. 16495356 10.1152/jn.01334.2005

[B12] Curtis C. E. Cole M. W. Rao V. Y. D’Esposito M. (2005). Canceling planned action: an FMRI study of countermanding saccades. *Cereb. Cortex* 15 1281–1289. 10.1093/cercor/bhi011 15616130

[B13] De Jong R. Coles M. G. Logan G. D. (1995). Strategies and mechanisms in nonselective and selective inhibitory motor control. *J. Exp. Psychol. Hum. Percept. Perform.* 21 498–511. 10.1037/0096-1523.21.3.498 7790830

[B14] De Jong R. Coles M. G. Logan G. D. Gratton G. (1990). In search of the point of no return: the control of response processes. *J. Exp. Psychol. Hum. Percept. Perform.* 16 164–182. 10.1037/0096-1523.16.1.164 2137517

[B15] Emeric E. E. Brown J. W. Boucher L. Carpenter R. H. Hanes D. P. Harris R. (2007). Influence of history on saccade countermanding performance in humans and macaque monkeys. *Vision Res.* 47 35–49. 10.1016/j.visres.2006.08.032 17081584 PMC1815391

[B16] Ethridge L. E. Soilleux M. Nakonezny P. A. Reilly J. L. Hill S. K. Keefe R. S. (2014). Behavioral response inhibition in psychotic disorders: diagnostic specificity, familiality and relation to generalized cognitive deficit. *Schizophr. Res.* 159 491–498. 10.1016/j.schres.2014.08.025 25261042 PMC4253557

[B17] Federico P. Mirabella G. (2014). Effects of probability bias in response readiness and response inhibition on reaching movements. *Exp. Brain Res.* 232 1293–1307. 10.1007/s00221-014-3846-8 24477763

[B18] Fuchs A. F. Kaneko C. R. Scudder C. A. (1985). Brainstem control of saccadic eye movements. *Annu. Rev. Neurosci.* 8 307–337. 10.1146/annurev.ne.08.030185.001515 3920944

[B19] Gauggel S. Rieger M. Feghoff T. A. (2004). Inhibition of ongoing responses in patients with Parkinson’s disease. *J. Neurol. Neurosurg. Psychiatry* 75 539–544. 15026491 10.1136/jnnp.2003.016469 PMC1739013

[B20] Godlove D. C. Emeric E. E. Segovis C. M. Young M. S. Schall J. D. Woodman G. F. (2011). Event-related potentials elicited by errors during the stop-signal task. I. Macaque monkeys. *J. Neurosci.* 31 15640–15649. 10.1523/JNEUROSCI.3349-11.2011 22049407 PMC3241968

[B21] Gulberti A. Arndt P. A. Colonius H. (2014). Stopping eyes and hands: evidence for non-independence of stop and go processes and for a separation of central and peripheral inhibition. *Front. Hum. Neurosci.* 8:61 10.3389/fnhum.2014.00061 PMC3927451 24600371

[B22] Hanes D. P. Patterson W. F. II Schall J. D. (1998). Role of frontal eye fields in countermanding saccades: visual, movement, and fixation activity. *J. Neurophysiol.* 79 817–834. 9463444 10.1152/jn.1998.79.2.817

[B23] Hanes D. P. Schall J. D. (1996). Neural control of voluntary movement initiation. *Science* 274 427–430. 10.1126/science.274.5286.427 8832893

[B24] Hanes D. P. Thompson K. G. Schall J. D. (1995). Relationship of presaccadic activity in frontal eye field and supplementary eye field to saccade initiation in macaque: poisson spike train analysis. *Exp. Brain Res.* 103 85–96. 10.1007/BF00241967 7615040

[B25] Harris M. S. Reilly J. L. Thase M. E. Keshavan M. S. Sweeney J. A. (2009). Response suppression deficits in treatment-naive first-episode patients with schizophrenia, psychotic bipolar disorder and psychotic major depression. *Psychiatry Res.* 170 150–156. 10.1016/j.psychres.2008.10.031 19906441 PMC2792232

[B26] Heimer L. (2012). *The Human Brain and Spinal Cord: Functional Neuroanatomy and Dissection Guide.* New York, NY: Springer-Verlag.

[B27] Hikosaka O. Sakamoto M. Usui S. (1989). Functional properties of monkey caudate neurons. I. Activities related to saccadic eye movements. *J. Neurophysiol.* 61 780–798. 2723720 10.1152/jn.1989.61.4.780

[B28] Jahfari S. Waldorp L. van den Wildenberg W. P. Scholte H. S. Ridderinkhof K. R. Forstmann B. U. (2011). Effective connectivity reveals important roles for both the hyperdirect (fronto-subthalamic) and the indirect (fronto-striatal-pallidal) fronto-basal ganglia pathways during response inhibition. *J. Neurosci.* 31 6891–6899. 10.1523/JNEUROSCI.5253-10.2011 21543619 PMC6632844

[B29] Jantz J. J. Watanabe M. Everling S. Munoz D. P. (2013). Threshold mechanism for saccade initiation in frontal eye field and superior colliculus. *J. Neurophysiol.* 109 2767–2780. 10.1152/jn.00611.2012 23486198

[B30] Kaneko C. R. (2006). Saccade-related, long-lead burst neurons in the monkey rostral pons. *J. Neurophysiol.* 95 979–994. 10.1152/jn.00760.2005 16236783 PMC1815387

[B31] Kornylo K. Dill N. Saenz M. Krauzlis R. J. (2003). Cancelling of pursuit and saccadic eye movements in humans and monkeys. *J. Neurophysiol.* 89 2984–2999. 10.1152/jn.00859.2002 12783947

[B32] Lappin J. S. Eriksen C. W. (1966). Use of a delayed signal to stop a visual reaction-time response. *J. Exp. Psychol.* 72 805–811. 10.1037/h0021266

[B33] Lauwereyns J. Watanabe K. Coe B. Hikosaka O. (2002). A neural correlate of response bias in monkey caudate nucleus. *Nature* 418 413–417. 10.1038/nature00892 12140557

[B34] Leigh R. J. Zee D. S. (2006). *The Neurology of Eye Movements.* New York, NY: Oxford University Press.

[B35] Leung H. C. Cai W. (2007). Common and differential ventrolateral prefrontal activity during inhibition of hand and eye movements. *J. Neurosci.* 27 9893–9900. 10.1523/JNEUROSCI.2837-07.2007 17855604 PMC6672638

[B36] Li C. S. Huang C. Constable R. T. Sinha R. (2006). Imaging response inhibition in a stop-signal task: neural correlates independent of signal monitoring and post-response processing. *J. Neurosci.* 26 186–192. 10.1523/JNEUROSCI.3741-05.2006 16399686 PMC6674298

[B37] Lipszyc J. Schachar R. (2010). Inhibitory control and psychopathology: a meta-analysis of studies using the stop signal task. *J. Int. Neuropsychol. Soc.* 16 1064–1076. 10.1017/S1355617710000895 20719043

[B38] Lisberger S. G. Fuchs A. F. (1978). Role of primate flocculus during rapid behavioral modification of vestibuloocular reflex. II. Mossy fiber firing patterns during horizontal head rotation and eye movement. *J. Neurophysiol.* 41 764–777. 96226 10.1152/jn.1978.41.3.764

[B39] Logan G. D. (1981). “Attention, automaticity, and the ability to stop a speeded choice response,” in *Attention and Performance IX*, eds Long J. Baddeley A. D. (Hillsdale, NJ: Erlbaum), 205–222.

[B40] Logan G. D. (1994). “On the ability to inhibit thought and action: a users’ guide to the stop signal paradigm,” in *Inhibitory Processes in Attention, Memory, and Language*, eds Dagenbach D. Carr T. H. (San Diego, CA: Academic Press), 189–239.

[B41] Logan G. D. Burkell J. (1986). Dependence and independence in responding to double stimulation: a comparison of stop, change, and dual-task paradigms. *Exp. Psychol. Hum. Percept. Perform.* 12 549–563.

[B42] Logan G. D. Cowan W. B. (1984). On the ability to inhibit thought and action: a theory of an act of control. *Psychol. Rev.* 91 295–327. 10.1016/j.neubiorev.2008.08.014 24490789

[B43] Logan G. D. Cowan W. B. Davis K. A. (1984). On the ability to inhibit simple and choice reaction time responses: a model and a method. *J. Exp. Psychol. Hum. Percept. Perform.* 10 276–291. 10.1037/0096-1523.10.2.276 6232345

[B44] Logan G. D. Irwin D. E. (2000). Don’t look! Don’t touch! Inhibitory control of eye and hand movements. *Psychon. Bull. Rev.* 7 107–112. 10.3758/BF03210728 10780023

[B45] Logan G. D. Schachar R. Tannock R. (1997). Impulsivity and inhibitory control. *Psychol. Sci.* 3 60–64. 10.1111/j.1467-9280.1997.tb00545.x

[B46] Luna B. Garver K. E. Urban T. A. Lazar N. A. Sweeney J. A. (2004). Maturation of cognitive processes from late childhood to adulthood. *Child Dev.* 75 1357–1372. 10.1111/j.1467-8624.2004.00745.x 15369519

[B47] Mattia M. Spadacenta S. Pavone L. Quarato P. Esposito V. Sparano A. (2012). Stop-event-related potentials from intracranial electrodes reveal a key role of premotor and motor cortices in stopping ongoing movements. *Front. Neuroeng.* 5:12 10.3389/fneng.2012.00012 PMC3386527 22754525

[B48] McDowell J. E. Dyckman K. A. Austin B. P. Clementz B. A. (2008). Neurophysiology and neuroanatomy of reflexive and volitional saccades: evidence from studies of humans. *Brain Cogn.* 68 255–270. 10.1016/j.bandc.2008.08.016 18835656 PMC2614688

[B49] Mink J. W. (1996). The basal ganglia: focused selection and inhibition of competing motor programs. *Prog. Neurobiol.* 50 381–425. 10.1016/S0301-0082(96)00042-1 9004351

[B50] Mirabella G. Iaconelli S. Romanelli P. Modugno N. Lena F. Manfredi M. (2012). Deep brain stimulation of subthalamic nuclei affects arm response inhibition in Parkinson’s patients. *Cereb. Cortex* 22 1124–1132. 10.1093/cercor/bhr187 21810782

[B51] Mirabella G. Pani P. Ferraina S. (2008). Context influences on the preparation and execution of reaching movements. *Cogn. Neuropsychol.* 25 996–1010. 10.1080/02643290802003216 19378414

[B52] Mirabella G. Pani P. Ferraina S. (2011). Neural correlates of cognitive control of reaching movements in the dorsal premotor cortex of rhesus monkeys. *J. Neurophysiol.* 106 1454–1466. 10.1152/jn.00995.2010 21697448

[B53] Mirabella G. Pani P. Pare M. Ferraina S. (2006). Inhibitory control of reaching movements in humans. *Exp. Brain Res.* 174 240–255. 10.1007/s00221-006-0456-0 16636792

[B54] Mosconi M. W. Kay M. D’Cruz A. M. Seidenfeld A. Guter S. Stanford L. D. (2009). Impaired inhibitory control is associated with higher-order repetitive behaviors in autism spectrum disorders. *Psychol. Med.* 39 1559–1566. 10.1017/S0033291708004984 19154646 PMC3145414

[B55] Nambu A. Tokuno H. Takada M. (2002). Functional significance of the cortico-subthalamo-pallidal ‘hyperdirect’ pathway. *Neurosci. Res.* 43 111–117. 10.1016/S0168-0102(02)00027-5 12067746

[B56] Ollman R. R. (1973). “Simple reactions with random countermanding of the go signal,” in *Attention and Performance IV*, ed. Kornblum S. (New York, NY: Academic Press), 571–582.

[B57] Oosterlaan J. Logan G. D. Sergeant J. A. (1998). Response inhibition in AD/HD, CD, comorbid AD/HD + CD, anxious, and control children: a meta-analysis of studies with the stop task. *J. Child Psychol. Psychiatry* 39 411–425. 9670096

[B58] Pare M. Hanes D. P. (2003). Controlled movement processing: superior colliculus activity associated with countermanded saccades. *J. Neurosci.* 23 6480–6489. 12878689 10.1523/JNEUROSCI.23-16-06480.2003 PMC6740637

[B59] Posner M. I. Snyder C. R. Davidson B. J. (1980). Attention and the detection of signals. *J. Exp. Psychol.* 109 160–174. 10.1037/0096-3445.109.2.160 7381367

[B60] Pouget P. Logan G. D. Palmeri T. J. Boucher L. Pare M. Schall J. D. (2011). Neural basis of adaptive response time adjustment during saccade countermanding. *J. Neurosci.* 31 12604–12612. 10.1523/JNEUROSCI.1868-11.2011 21880921 PMC3173043

[B61] Reinhart R. M. Carlisle N. B. Kang M. S. Woodman G. F. (2012). Event-related potentials elicited by errors during the stop-signal task. II: human effector-specific error responses. *J. Neurophysiol.* 107 2794–2807. 10.1152/jn.00803.2011 22357790 PMC3362284

[B62] Rieger M. Gauggel S. (1999). Inhibitory after-effects in the stop signal paradigm. *Br. J. Psychol.* 90 509–518. 10.1348/000712699161585

[B63] Scangos K. W. Stuphorn V. (2010). Medial frontal cortex motivates but does not control movement initiation in the countermanding task. *J. Neurosci.* 30 1968–1982. 10.1523/JNEUROSCI.4509-09.2010 20130204 PMC4041090

[B64] Schall J. D. (2002). The neural selection and control of saccades by the frontal eye field. *Philos. Trans. R. Soc. Lond. B Biol. Sci.* 357 1073–1082. 10.1098/rstb.2002.1098 12217175 PMC1693021

[B65] Schall J. D. Hanes D. P. Taylor T. L. (2000). Neural control of behavior: countermanding eye movements. *Psychol. Res.* 63 299–307. 10.1007/s004269900008 11004883

[B66] Schouten J. F. Bekker J. A. (1967). Reaction time and accuracy. *Acta Psychol.* 27 143–153. 10.1016/0001-6918(67)90054-6 6062205

[B67] Stuphorn V. Brown J. W. Schall J. D. (2010). Role of supplementary eye field in saccade initiation: executive, not direct, control. *J. Neurophysiol.* 103 801–816. 10.1152/jn.00221.2009 19939963 PMC2822692

[B68] Verbruggen F. Logan G. D. (2008). After-effects of goal shifting and response inhibition: a comparison of the stop-change and dual-task paradigms. *Q. J. Exp. Psychol. (Hove)* 61 1151–1159. 10.1080/17470210801994971 18938760

[B69] Verbruggen F. Logan G. D. (2009). Proactive adjustments of response strategies in the stop-signal paradigm. *J. Exp. Psychol. Hum. Percept. Perform.* 35 835–854. 10.1037/a0012726 19485695 PMC2690716

[B70] Verfaellie M. Heilman K. M. (1987). Response preparation and response inhibition after lesions of the medial frontal lobe. *Arch. Neurol.* 44 1265–1271. 10.1001/archneur.1987.00520240045010 3675260

[B71] Vince M. (1948). The intermittency of control movements and the psychological refractory period. *Br. J. Psychol.* 38 149–157. 10.1111/j.2044-8295.1948.tb01150.x 18913658

[B72] Wechsler D. (1999). *Wechsler Abbreviated Scale of Intelligence.* New York, NY: The Psychological Corporation.

[B73] Wijnen J. G. Ridderinkhof K. R. (2007). Response inhibition in motor and oculomotor conflict tasks: different mechanisms, different dynamics? *Brain Cogn.* 63 260–270. 10.1016/j.bandc.2006.09.003 17069944

